# The *p* factor outweighs the specific internalizing factor in predicting recurrences of adolescent depression

**DOI:** 10.1192/j.eurpsy.2024.18

**Published:** 2024-03-01

**Authors:** Yinuo Shu, Na Ao, Xue Wen, Zaixu Cui, Diyang Qu, Runsen Chen

**Affiliations:** 1 Chinese Institute for Brain Research, Beijing, China; 2 Vanke School of Public Health, Tsinghua University, Beijing, China; 3 Institute for Healthy China, Tsinghua University, Beijing, China

**Keywords:** adolescence, depression, *p* factor, psychopathology factor, recurrence

## Abstract

**Background:**

The early prediction of adolescent depression recurrence poses a significant challenge in the field. This study aims to investigate and compare the abilities of the general psychopathology factor (*p*) and the specific internalizing factor, in predicting depression recurrence over a 2-year course, as well as identifying remitted depressed adolescents from healthy adolescents. Longitudinal changes of these two factors in different trajectory groups were also tracked to examine their sensitivity to sustained remission and relapse.

**Methods:**

We included 255 baseline-remitted depressed adolescents and a healthy control group (*n* = 255) matched in age, sex, and race, sourced from the Adolescent Brain Cognitive Development Study. The linear mixed model was employed for the statistical analysis.

**Results:**

The *p* factor not only effectively discriminated between remitted depressed adolescents and healthy controls but also robustly predicted the depression recurrence over a subsequent 2-year course. The specific internalizing factor could only differentiate remitted depressed adolescents from healthy controls. Additionally, a noteworthy longitudinal decline of the *p* factor in the sustained-remission group was observed.

**Conclusions:**

Psychopathology factors serve as the inherent and enduring measurement of long-term mental health aberrations. Longitudinal results indicate that the *p* factor is more sensitive to respond to sustained remission than the internalizing factor. The ability of the overall *p* factor to anticipate depression relapse, unlike the specific internalizing factor, suggests the clinical interventions should monitor and mitigate the coincident symptoms across all dimensions to preempt relapse of adolescent depression, rather than an exclusive focus on internalizing symptoms.

## Introduction

Depressive disorders, as an umbrella term, ranging from major depression to atypical depression to dysthymia, are one of the most serious mental health concerns and leading contributors of the global health-related burden [1–4]. Depression is more common in adolescents than in prepubertal children [5]. Adolescents are particularly at risk for developing depression, with estimates of major depressive disorder ranging from 8% to 20% occurring before the age of 18 [5–9]. In addition, depression frequently co-occurs with anxiety in adolescents, occurring both simultaneously and sequentially, and the emergence of depression often heightens the risk of developing anxiety over the course of time [10].

Notably, depression that begins in adolescence often presents as a recurring condition, with a higher risk of recurrence linked to an older onset age [11]. The recurrence rate ranges from 20% to 54%, and this form of depression tends to be associated with more severe outcomes when compared to depression that begins in adulthood [12–15]. This recurrent pattern can lead to substantial impairments across crucial psychosocial domains, with effects that may persist into adulthood [16–19]. However, there is no permanent treatment solution for depressive disorders due to their relapsing–remitting nature. Individuals who experience a relapse after the treatment of their first episode of depression may tend to recur with greater severity and with lessening responsivity to conventional treatments [20]. Therefore, there is a pressing need for a deeper understanding of early-stage markers that can predict the later development of depression recurrence [18, 21–23]. Monitoring changes in markers can help individuals and healthcare professionals become aware of early warning signs of a potential relapse. This awareness allows them to implement tailored interventions, enhancing the effectiveness of relapse prevention and even circumventing the onset of initial treatment resistance. In another word, this approach may heighten the prospect of sustained recovery and prevent the intensification of depression during the later stages of adulthood [24].

Prior research has identified various risk factors for depression recurrence in remitted patients, including a higher number of preceding episodes, higher levels of residual symptoms, lower levels of positive refocusing [25], presence of anxiety [26], longer symptom duration, higher symptom severity, and earlier age of onset [27]. While previous studies have made valuable contributions to our understanding of predictors for depression recurrence, a shared limitation is their focus on syndrome-specific indicators, such as the Patient Health Questionnaire-9 or the Hamilton Depression Rating Scale, designed to detect specific signs or symptoms of depression. However, considering the latent intricate etiology of depression, which encompasses interplays of a broader spectrum of symptoms across multiple dimensions and a high degree of comorbidity with other disorders like externalizing disorders that can significantly impact the trajectory of depression and increase the risk of further recurrence [28], the incorporation of multidimensional psychopathology becomes a necessity. Therefore, using the indicators that encompass information from symptoms across multiple dimensions could potentially yield a more accurate prediction of depression recurrence. Recent advancements in psychopathology studies have indicated an overall latent factor, the general psychopathology factor, that may further provide an explanation for these pathways [29].

The general psychopathology factor, commonly referred to as the *p* factor, accounts for common variance across a wide range of symptoms spanning multiple diagnostic domains [30]. It embodies shared aspects among various mental disorders [31] and directly impacts symptoms across distinct dimensions [32]. Previous research has identified the presence of the *p* factor in adolescents and suggested that investigating this factor could enhance our understanding of the etiology, risk, and correlates of psychopathology in this age group [31, 33, 34]. For instance, Moore et al. [35] identified the overall *p* factor through a bi-factor model using the Child Behavior Checklist (CBCL) from the Adolescent Brain Cognitive Development (ABCD) Study. In this bi-factor model, three lower-level factors in distinct domains (internalizing, Attention Deficit Hyperactivity Disorder [ADHD], and conduct problems) have also been identified, which account for shared variance within a specific dimension, from which the overall variance (*p*) across all dimensions has been subtracted.

Indeed, compared to previously identified predictors such as diagnostic comorbidity at baseline, a recent study suggested that *p* at baseline in adolescents with anxiety predicted long-term anxiety outcomes, including more mental health disorders, poorer functioning, and greater impairment, more effectively [36]. In addition, as compared to syndrome-specific psychopathology factors, another recent study has found that the *p* factor in adolescents may be more predictive of long-term adverse mental health outcomes, including diagnoses of depression and anxiety, psychological well-being, criminal activity, alcohol use, and educational attainment. This finding indicates that interventions should focus on addressing the co-occurrence of internalizing and externalizing symptoms to mitigate the long-term impact on individuals [33]. However, there is limited knowledge regarding the prognostic effect of the overall *p* factor or the specific lower-level internalizing factor to which depression is directly related on the prediction of adolescent depression recurrences. Additionally, the comparative performance of these two factors in terms of their predictive ability in depression recurrence remains unknown. This information would be valuable in guiding interventions and improving the effectiveness of targeted treatments for depression in adolescents.

To fill this research gap, our study evaluated the capacity of the two factors – the *p* factor and the specific internalizing factor – all measured during a remitted state, to predict the recurrence of depression over a 2-year period, and to discriminate between depressed adolescents and their healthy counterparts. This was achieved by using two waves of clinical data collected at baseline and at a 2-year follow-up from the ABCD Study. Simultaneously, we tracked the longitudinal change of each factor over 2 years to detect their sensitivity in response to either sustained remission or relapse. Our aim is to enhance our understanding of the underlying mechanisms involved in the recurrence of adolescent depression and provide valuable insights into effective intervention strategies for managing recurrent depression in adolescents.

## Methods

### Participants

Data for this study were derived from a large-scale, multi-site, and longitudinal study in the United States: the ABCD Study® (Release 3.0, November 2020) [37]. This extensive dataset included comprehensive clinical, behavioral, cognitive, and multimodal neuroimaging data collected at four distinct timepoints (baseline, 1-year follow-up, 2-year follow-up, and 3-year follow-up). The current research focused on a portion of the baseline data (*n* = 11,876, aged 9–10 years) and the 2-year follow-up data (*n* = 10,404, aged 11–12 years) within the ABCD Study, given that Kiddie Schedule for Affective Disorders and Schizophrenia (KSADS) depressive diagnostic information was collected biennially.

**Figure 1.**
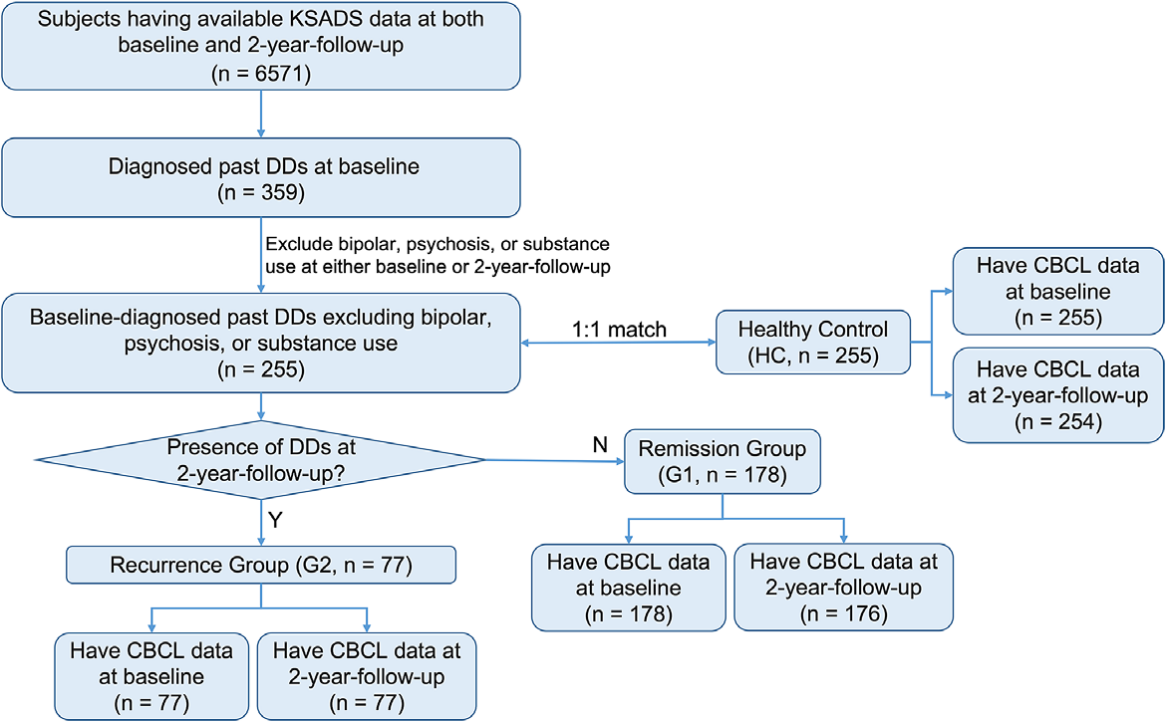
Flowchart of participant selection criteria and group allocation. DDs, depressive disorders, including major depressive disorder, dysthymia, and unspecified depressive disorders; Y, yes; N, no; *n*, the number of participants.

Depressive disorder diagnoses were determined using parent or guardian ratings in the computerized KSADS based on the Diagnostic and Statistical Manual of Mental Disorders, Fifth Edition criteria [38]. Our study included 255 subjects who met our selection criteria (Figure 1): (i) presence of a diagnosed past major depressive disorder (MDD), dysthymia, or unspecified depressive disorder at baseline and (ii) exclusion of a diagnosed bipolar disorder, psychosis, or substance use at either baseline or 2-year follow-up. It should be noted that our study concentrated on subjects who received KSADS diagnoses of past (in a remitted state at the moment of baseline measurement) depressive disorders at the baseline. This was due to the limited number of subjects diagnosed with present depressive disorders at both timepoints.

The current study also involved a control group of 255 healthy individuals (HC, *M*_age_ = 118.41 months, SD = 7.20; 53.73% were girls, and 50.98% were non-Hispanic White) who showed none of any KSADS diagnoses at both the baseline and the 2-year follow-up. These control subjects were matched with the 255 depressed adolescents on age, sex, and race. We have also tested all subsequent statistical analyses utilizing the unmatched healthy control sample (*n* = 1,597), and the outcomes were consistent with those derived from the matched healthy controls (Supplementary Table 2 and Supplementary Figure 1).

### Definition of diagnosis trajectory groups

We defined the diagnosis trajectory groups according to the absence (G1, *n* = 178; *M*_age_ = 119.23 months, SD = 7.24; 44.38% were girls and 51.69% were non-Hispanic White) or presence (G2, *n* = 77; *M*_age_ = 121.38 months, SD = 7.74; 49.35% were girls and 61.04% were non-Hispanic White) of 2-year follow-up parent-report KSADS diagnosed MDD, dysthymia, or unspecified depressive disorder that was present (in the recent 2 weeks, *n* = 4), in partial remission (*n* = 4), or past (since baseline assessment, *n* = 75) (Figure 1). According to the defined criteria, G1 represented a remission group with participants who experienced no recurrence of depression for a minimum of 2 years, while G2 represented a recurrence group consisting of individuals who were in remission at the baseline measurement but experienced a recurrence over the subsequent 2-year period. Thus, G1 represented a more favorable trajectory, tending toward stable remission, while G2 represented a recurrence trajectory within the 2-year course.

### Demographic information

Age, sex, race, and site were included as covariates (Supplementary Table 1).

### Measure of psychopathology

Psychopathology was measured from parent-reported CBCL, which was used to assess emotional and behavioral problems in school-aged children [39]. All 119 items were scored using a 3-point Likert scale, ranging from 0 (“not true”) to 2 (“very true”). In a prior study [35], exploratory factor analyses of the CBCL data were initially conducted using a random half of the ABCD Study sample (*N* = 5,932). Among the various models, the bi-factor model identified four distinct psychopathology factors – comprising the general psychopathology factor (*p* factor) and three subordinate factors: internalizing, ADHD, and conduct problems. These factors exhibited significant associations with external criterion measures. In our current study, we constructed the bi-factor confirmatory model based on the entire ABCD sample at both baseline (*N* = 11,866) and the 2-year follow-up (*N* = 10,353), using the confirmed exploratory model structure derived from the prior study [35]. Our models demonstrated a good fit, meeting conventional fit thresholds (baseline model: *χ* ^2^ = 17,611.842, *p* < 0.001; RMSEA = 0.026; CFI = 0.936; TLI = 0.931; SRMR = 0.060; follow-up model: *χ* ^2^ = 14,928.909, *p* < 0.001; RMSEA = 0.025; CFI = 0.930; TLI = 0.926; SRMR = 0.064).

### Statistical analysis

In our present statistical analysis, we intentionally concentrated solely on the overall *p* factor and the internalizing factor, omitting the lower-level psychopathology factors associated with ADHD and conduct problems dimensions. This deliberate exclusion aligns with the study’s primary aim, which centers on discerning the differences in predicting depression trajectories between the overall *p* factor and the specific lower-level factor encompassing depression.

#### Predict depression trajectories

Generalized linear mixed model (GLMM) was applied to predict depression trajectory groups utilizing R lmeTest packages [40]. For the two trajectory groups (G1 and G2), the ability of the baseline *p* factor and baseline specific internalizing factor, respectively, in predicting trajectory groups were examined. In the GLMM formula, the *p* factor and the specific internalizing factor served as the independent variable respectively, the group was the dependent variable, and age, sex, and race were employed as fixed-effects covariates, while site was utilized as a random-effects covariate [41].

In addition, we have also examined the predictive effects of the *p* factor and the internalizing factor on depression recurrences while controlling for each other, in order to further elucidate the distinct information contributed by each in predicting depression recurrences when the other is controlled. To accomplish this, both the *p* factor and the internalizing factor were concurrently incorporated into the GLMM formula to forecast the dependent variable “group.” Fixed-effects covariates included age, sex, and race, whereas site served as a random-effects covariate.

All the continuous variables were standardized, including the *p* factor, the specific internalizing factor, and age. Other categorical variables were dummy coded before being put into the model, including group, sex, race, and site [42]. The false discovery rate (FDR) was applied for multiple comparisons to avoid type I errors.

#### Distinguish remitted depressed adolescents from healthy controls

The same GLMM model was used to distinguish between depressed adolescents and healthy controls, only differing in that the dependent variable “group” was either HC and G1 or HC and G2. FDR was applied for multiple comparisons.

#### Longitudinal analysis

The longitudinal alterations of the *p* factor and the specific internalizing factor in each group were investigated by employing linear mixed model. For each group, the *p* factor and the specific internalizing factor functioned as the dependent variable respectively, the time variable (baseline defined as 0, 2-year follow-up defined as 1) served as the independent variable, and sex and race were used as fixed-effects covariates, with site as a random-effects covariate. Additionally, the participant was incorporated as a random-effects covariate to eliminate individual differences. FDR was applied for multiple comparisons.

## Results

### Psychopathology factors distinguish remitted depressed adolescents from healthy controls

**Figure 2.**
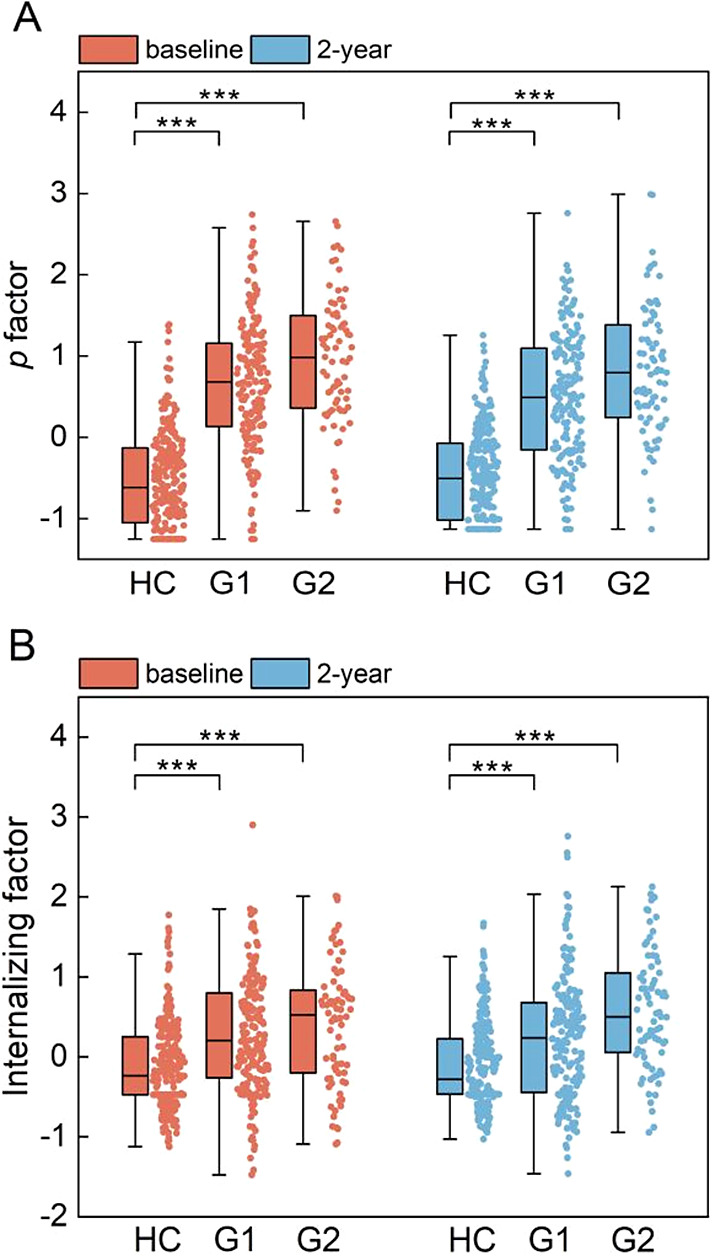
Results of the *p* factor and internalizing factor distinguishing between remitted depressed adolescents and healthy controls at baseline and 2-year follow-up. (A) The distribution of the *p* factor in each subgroup at both baseline and 2-year follow-up measurements. (B) The distribution of the specific internalizing factor in each subgroup at both baseline and 2-year follow-up measurements. HC, healthy group; G1, remission group; G2, recurrence group. ***p < 0.001; **p < 0.01.

Both the *p* factor and the internalizing factor were capable of distinguishing depressed adolescents from healthy controls, as both G1 and G2 exhibited significantly higher *p* factor than HC at both baseline (HC vs. G1: *p*_FDR_ < 0.0001, *β* = 2.12; HC vs. G2: *p*_FDR_ < 0.0001, *β* = 2.71) and 2-year follow-up (HC vs. G1: *p*_FDR_ < 0.0001, *β* = 1.69; HC vs. G2: *p*_FDR_ < 0.0001, *β* = 2.48) (Figure 2A) as well as higher internalizing factor than HC at both baseline (HC vs. G1: *p*_FDR_ < 0.0001, *β* = 0.69; HC vs. G2: *p*_FDR_ < 0.0001, *β* = 0.85) and 2-year follow-up (HC vs. G1: *p*_FDR_ < 0.0001, *β* = 0.58; HC vs. G2: *p*_FDR_ < 0.0001, *β* = 1.14). The boxplots of the *p* factor and the internalizing factor in each subgroup at baseline and 2-year follow-up are presented in Figure 2B. Detailed modeling results are shown in Table 1.

**Table 1.** Generalized linear mixed modeling results for using psychopathology factors to distinguish depressed adolescents from HC and predict future depression trajectories after controlling for age, sex, race, and site

Factors	Use psychopathology factors to distinguish depressed adolescents from HC												Use baseline psychopathology factors to predict future depression trajectories		
	HC vs. G1						HC vs. G2						G1 vs. G2		
	Baseline			2-year			Baseline			2-year			Baseline		
	*β*	*SE*	*p_FDR_*	*β*	*SE*	*p_FDR_*	*β*	*SE*	*p_FDR_*	*β*	*SE*	*p_FDR_*	*β*	*SE*	*p_FDR_*
*p* factor	2.12	0.21	3.54 × 10^−23^	1.69	0.18	5.35 × 10^−20^	2.71	0.34	1.10 × 10^−14^	2.48	0.32	3.99 × 10^−14^	0.44	0.15	0.0041
Internalizing factor	0.69	0.11	1.77 × 10^−9^	0.58	0.11	3.73 × 10^−7^	0.85	0.15	9.56 × 10^−9^	1.14	0.16	4.24 × 10^−12^	0.19	0.14	0.20

*Note*: *β*, standardized coefficient; SE, standard error; *p*_*FDR*_, *p* values after FDR.

### The p factor predicts depression trajectories

**Figure 3.**
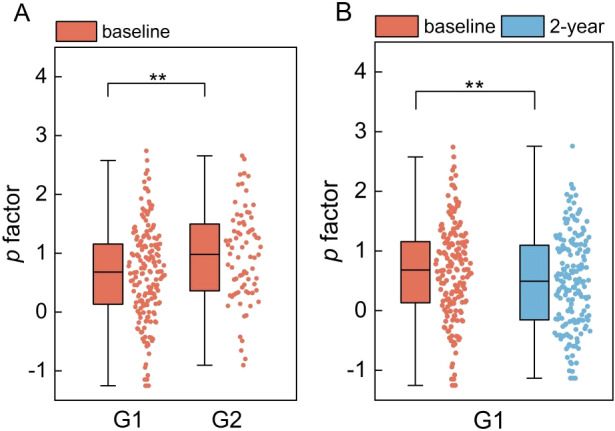
Results of the *p* factor predicting depression trajectories and mirroring sustained remission over a 2-year course. (A) The results that baseline *p* factor in the recurrence group were significantly higher than baseline *p* factor in the remission group. (B) The longitudinal decrease of the *p* factor in the remission group from baseline to 2-year follow-up. G1, remission group; G2, recurrence group. ***p < 0.001; **p < 0.01.

Of the two factors examined, only the baseline *p* factor was found to be capable of predicting depression trajectories over the subsequent 2-year period (G1 vs. G2: *p*_FDR_ < 0.01, *β* = 0.44), with a higher *p* factor being indicative of depression recurrence (Figure 3A). However, the baseline internalizing factor failed to exhibit predictive ability in predicting depression trajectories over the next 2 years (G1 vs. G2: *p*_FDR_ > 0.05, *β* = 0.19). Detailed modeling results are shown in Table 1.

Furthermore, it was observed that the baseline *p* factor consistently and significantly predicted depression recurrences (*p_FDR_* < 0.01, *β* = 0.44), even when accounting for the baseline internalizing factor. In contrast, the baseline internalizing factor exhibited a weak and non-significant association with depression recurrences (*p_FDR_* > 0.05, *β* = 0.18).

### Longitudinal decrease of the p factor in the remission group

Among the three groups, only G1 exhibited a significant decrease in *p* factor from baseline to 2-year follow-up (*p*_FDR_ < 0.01, *β* = 0.22) (Figure 3B). And no significant changes of the specific internalizing factor were found in any of the three groups. Detailed modeling results are shown in Table 2.

**Table 2.** Linear mixed modeling results of longitudinal analysis after controlling for age, sex, race, and site

Group	*p* factor			Internalizing factor		
	*β*	*SE*	*p_FDR_*	*β*	*SE*	*p_FDR_*
HC	−0.098	0.058	0.092	−0.047	0.073	0.52
G1	0.22	0.067	0.0041	0.056	0.070	0.52
G2	0.20	0.10	0.081	−0.20	0.10	0.14

*Note*: *β*, standardized coefficient; SE, standard error; *p_FDR_*, *p* values after FDR.

## Discussion

This study aims to examine and compare the impact of the specific internalizing factor and the *p* factor on the prediction of depression recurrence, as well as their ability to differentiate between currently remitted depressed adolescents and healthy individuals. Our research findings reveal that latent psychopathology factors may serve as inherent and enduring indicators for long-term mental health aberrations, whereas the *p* factor, rather than the internalizing factor, exhibits sensitivity to both relapse and sustained remission of depression. Therefore, it is crucial to emphasize the importance of monitoring and intervening in the co-occurrence of symptoms across all dimensions, represented by the *p* factor, as an effective tool to prevent the recurrence of adolescent depression.

We first assessed the ability of the specific internalizing factor and *p* factor, respectively, in distinguishing currently remitted depressed adolescents from their healthy counterparts. Our results revealed that both the specific internalizing factor and *p* factor were found to be significantly higher in depressed adolescents compared to healthy controls even if measured at a remitted state. This observation indicates that both the overall *p* factor and the specific internalizing factor are capable of capturing residual symptoms in remitted depressed adolescents, and are therefore greatly equipped to identifying remitted depressed adolescents. And this finding is also consistent with views common in the antidepressant field that the depressive disorders are merely suppressed at a remitted state and the underlying disturbance continues until spontaneous remission occurs [43], which explains why the remitted patients exhibited higher specific internalizing factor and *p* factor than their healthy counterparts.

Subsequently, we examined the prognostic effect of the two factors on depression recurrence separately. Our study specifically found that an elevated baseline *p* factor (not the internalizing factor), measured during a remitted state, can effectively predict the recurrence of depression in the following 2 years, even with the information of the specific internalizing factor controlled. This observation aligns with a recent study that discovered patients with a high baseline *p* factor were more likely to experience poorer outcomes in terms of short-term psychotherapy response [44]. This observation reflects the relatively insensitive or insubstantial role of the specific internalizing factor in predicting depression recurrence. In contradistinction, the overarching *p* factor presents itself as a more sensitive predictor, aligning with prior research that indicated the *p* factor’s greater relevance for long-term outcomes compared to specific factors [33].

It is important to note that our findings do not imply that internalizing symptoms have no association with future depression recurrence. In fact, our results suggest that once the shared variance across all dimensions (the *p* factor) is taken into account, the remaining unique variance (the specific internalizing factor) does not significantly relate to future depression recurrence [33]. This observation highlights the crucial role of symptoms in other dimensions of psychopathology, such as ADHD and conduct problems, in the development of future episodes. In line with the transdiagnostic approach, the findings lead us to consider recurrent depression as potentially arising from complex interactions among symptoms across all dimensions, rather than being strictly confined to the internalizing dimension [28, 45]. By acknowledging the intricate interplay of symptoms across diverse dimensions, we gain a deeper understanding of the complex nature of recurrent depression and its underlying mechanisms. This realization prompts a shift in perspective, which highlights the importance of monitoring and addressing comorbid symptoms across all dimensions [46]. In this process, the *p* factor emerges as a highly sensitive tool for detecting the risk of future depression recurrences [32]. Further research is warranted to delve deeper into the mechanisms underlying the influence of specific psychopathology factors, especially given that the total variance of the *p* factor has been accounted for [29].

Furthermore, even after an extended period of remission (at least 2 years), both the specific internalizing factor and the *p* factor in the remission group remained considerably higher than that of the healthy controls at the 2-year follow-up measurement. This observation suggests that individuals with a history of depression might continue to exhibit a higher specific internalizing factor and *p* factor compared to those without such a history, even during prolonged, stable recovery and when deemed healthy at the time of assessment. This observation indicates that latent psychopathology factors reflect inherent and enduring mental health deviations, which may serve as a straightforward and effective measurement for lifetime psychopathology evaluations.

Interestingly, the longitudinal analysis over 2 years showed a significant decrease in the *p* factor within the remission group. However, no significant longitudinal changes were found in the specific internalizing factor. This observation aligns with a previous study that observed a diminishing pattern in the *p* factor during short-term psychotherapies, while the specific lower-level factors remained stable [44]. This finding suggests that the sustained depression remission may not necessarily induce significant changes in the specific characteristics of the internalizing dimension when *p*’s variance was accounted for. In contrast, the fluctuation of the *p* factor is more sensitive to reflect sustained remission. Therefore, the significant decline in the *p* factor could be considered as a more sensitive and effective indicator for detecting the long-term remission of depression compared to the lower-level specific internalizing factor.

Several limitations inherent to our study should be acknowledged. First, this study was only based on parent-reported KSADS diagnoses because of the lack of child-reported KSADS diagnosis data of depressive disorders in the ABCD release 3.0. Controversy exits in previous findings about the discrepancies between parent- and child-reports of depression. Some studies found significant but low agreement between parents and their children about depressive symptoms [47]. However, some studies suggested that discrepancies between informants were not clinically meaningful [48]. Future studies are needed to validate our findings using both child- and parent-reported diagnoses and examine the concordance of results from different informants. Second, the delineation of depression trajectories in our study was based on KSADS diagnoses at two timepoints. While informative, this approach may not capture the full nuance of depression trajectories. Future research should aim to explore more precisely defined depression trajectories. Third, we were unable to consider factors such as first onset age, number of episodes, and antidepressant treatment due to the lack of available data from the ABCD cohort. It would be valuable for subsequent studies to explore the influence of these factors on predicting depression recurrence. Despite these limitations, our study provides valuable insights and lays the groundwork for further research in this area.

## Conclusion

In conclusion, our study sheds light on the critical role of the *p* factor, rather than the specific internalizing factor, in predicting future recurrence of adolescent depression and mirroring sustained remission. Moreover, our study suggested the importance of monitoring and intervening in the co-occurrence of symptoms across all dimensions in preventing adolescent depression recurrence, rather than solely focusing on the internalizing dimension. Further research examining the role of the *p* factor in predicting adolescent depression trajectories over an extended period and investigating novel interventions and treatments aimed at mitigating symptoms across all dimensions and reducing the *p* factor could be conducted.

## Supporting information

Shu et al. supplementary material Shu et al. supplementary material

## Supplementary material

The supplementary material for this article can be found at http://doi.org/10.1192/j.eurpsy.2024.18.

## References

[1] Smith K. Mental health: a world of depression. Nature. 2014;515:180–1. 10.1038/515180a 25391942

[2] Friedrich MJ. Depression is the leading cause of disability around the world. JAMA. 2017 Apr 18;317(15):1517. 10.1001/jama.2017.3826. 28418490

[3] Herrman H, Patel V, Kieling C, Berk M, Buchweitz C, Cuijpers P, et al. Time for united action on depression: a lancet–world psychiatric association commission. Lancet. 2022 Mar;399(10328):957–1022. 10.1016/S0140-6736(21)02141-3. 35180424

[4] Marcus M, Yasamy MT, Van Ommeren MV, Chisholm D, Saxena S. Depression: a global public health concern. 2012. 10.1037/e517532013-004.

[5] Thapar A, Collishaw S, Pine DS, Thapar AK. Depression in adolescence. Lancet. 2012 Mar 17;379(9820):1056–67. 22305766 10.1016/S0140-6736(11)60871-4 PMC3488279

[6] Cheung A, Dewa C. Canadian community health survey: major depressive disorder and suicidality in adolescents. Healthc Policy. 2006 Nov 15;2(2):76–89. 10.12927/hcpol.2007.18540. 19305706 PMC2585433

[7] Hankin BL, Abramson LY, Moffitt TE, Silva PA, McGee R, Angell KE. Development of depression from preadolescence to young adulthood: emerging gender differences in a 10-year longitudinal study. J Abnorm Psychol. 1998;107(1), 128–40. 10.1037/0021-843X.107.1.128. 9505045

[8] Kessler RC, Walters EE. Epidemiology of DSM-III-R major depression and minor depression among adolescents and young adults in the national comorbidity survey. Depress Anxiety. 1998;7(1):3–14. 10.1002/(SICI)1520-6394(1998)7:1<3::AID-DA2>3.0.CO;2-F. 9592628

[9] Naicker K, Galambos NL, Zeng Y, Senthilselvan A, Colman I. Social, demographic, and health outcomes in the 10 years following adolescent depression. J Adolesc Health. 2013 May;52(5):533–8. 10.1016/j.jadohealth.2012.12.016. 23499382

[10] Garber J, Weersing VR. Comorbidity of anxiety and depression in youth: implications for treatment and prevention. Clin Psychol Sci Pract. 2010 Dec;17(4):293. 10.1111/j.1468-2850.2010.01221.x PMC3074295 21499544

[11] Birmaher B, Williamson DE, Dahl RE, Axelson DA, Kaufman J, Dorn LD, Ryan ND. Clinical presentation and course of depression in youth: Does onset in childhood differ from onset in adolescence? J Am Acad Child Adolesc Psychiatry. 2004 Jan 1;43(1):63–70. 14691361 10.1097/00004583-200401000-00015

[12] Birmaher B. Course and outcome of child and adolescent major depressive disorder. Child Adolesc Psychiatr Clin N Am. 2002 Jul;11(3):619–37. 10.1016/S1056-4993(02)00011-1. 12222086

[13] Fombonne E, Wostear G, Cooper V, Harrington R, Rutter M. The Maudsley long-term follow-up of child and adolescent depression: I. Psychiatric outcomes in adulthood. Br J Psychiatry. 2001 Sep;179(3):210–7. 10.1192/bjp.179.3.210. 11532797

[14] Lewinsohn PM, Rohde P, Klein DN, Seeley JR. Natural course of adolescent major depressive disorder: I. Continuity into young adulthood. J Am Acad Child Adolesc Psychiatry. 1999 Jan;38(1):56–63. 10.1097/00004583-199901000-00020. 9893417

[15] Weissman MM. Depressed adolescents grown up. JAMA. 1999 May 12;281(18):1707. 10.1001/jama.281.18.1707. 10328070

[16] Brière FN, Rohde P, Seeley JR, Klein D, Lewinsohn PM. Comorbidity between major depression and alcohol use disorder from adolescence to adulthood. Compr Psychiatry. 2014 Apr;55(3):526–33. 10.1016/j.comppsych.2013.10.007. 24246605 PMC4131538

[17] Clayborne ZM, Varin M, Colman I. Systematic review and meta-analysis: adolescent depression and long-term psychosocial outcomes. J Am Acad Child Adolesc Psychiatry. 2019 Jan;58(1):72–9. 10.1016/j.jaac.2018.07.896. 30577941

[18] Johnson D, Dupuis G, Piche J, Clayborne Z, Colman I. Adult mental health outcomes of adolescent depression: a systematic review. Depress Anxiety. 2018 Aug;35(8):700–16. 10.1002/da.22777. 29878410

[19] Wilson S, Dumornay NM. Rising rates of adolescent depression in the United States: challenges and opportunities in the 2020s. J Adolesc Health. 2022 Mar;70(3):354–5. 10.1016/j.jadohealth.2021.12.003. 35183317 PMC8868033

[20] Ali S, Rhodes L, Moreea O, McMillan D, Gilbody S, Leach C, et al. How durable is the effect of low intensity CBT for depression and anxiety? Remission and relapse in a longitudinal cohort study. Behav Res Ther. 2017 Jul;94:1–8. 10.1016/j.jadohealth.2021.12.003. 28437680

[21] Burcusa SL, Iacono WG. Risk for recurrence in depression. Clin Psychol Rev. 2007 Dec;27(8):959–85. 10.1016/j.cpr.2007.02.005. 17448579 PMC2169519

[22] Colizzi M, Lasalvia A, Ruggeri M. Prevention and early intervention in youth mental health: Is it time for a multidisciplinary and trans-diagnostic model for care? Int J Ment Health Syst. 2020 Dec;14(1):23. 10.1186/s13033-020-00356-9. 32226481 PMC7092613

[23] Davey CG, McGorry PD. Early intervention for depression in young people: a blind spot in mental health care. Lancet Psychiatry. 2019 Mar;6(3):267–72. 10.1016/S2215-0366(18)30292-X. 30502077

[24] Dinga R, Marquand AF, Veltman DJ, Beekman ATF, Schoevers RA, van Hemert AM, et al. Predicting the naturalistic course of depression from a wide range of clinical, psychological, and biological data: A machine learning approach. Transl Psychiatry. 2018 Nov 5;8(1):241. 10.1038/s41398-018-0289-1. 30397196 PMC6218451

[25] ten Doesschate MC, Bockting CL, Koeter MW, Schene AH, DELTA Study Group. Prediction of recurrence in recurrent depression: a 5.5-year prospective study. J Clin Psychiatry. 2010;71(8):984–91. 20797379 10.4088/JCP.08m04858blu

[26] Penninx BWJH, Nolen WA, Lamers F, Zitman FG, Smit JH, Spinhoven P, et al. Two-year course of depressive and anxiety disorders: results from the Netherlands study of depression and anxiety (NESDA). J Affect Disord. 2011 Sep;133(1–2):76–85. 10.1016/j.jad.2011.03.027. 21496929

[27] Pettit JW, Lewinsohn PM, Roberts RE, Seeley JR, Monteith L. The long-term course of depression: development of an empirical index and identification of early adult outcomes. Psychol Med. 2009 Mar;39(3):403–12. 10.1017/S0033291708003851. 18606049 PMC2744453

[28] Wolff JC, Ollendick TH. The comorbidity of conduct problems and depression in childhood and adolescence. Clin Child Fam Psychol Rev 2006 Dec 8;9(3–4):201–20. 10.1007/s10567-006-0011-3. 17053962

[29] Smith GT, Atkinson EA, Davis HA, Riley EN, Oltmanns JR. The general factor of psychopathology. Annu Rev Clin Psychol. 2020;16:75–98. 10.1146/annurev-clinpsy-071119-115848. 32040926

[30] Murray AL, Eisner M, Ribeaud D. The development of the general factor of psychopathology “*p* factor” through childhood and adolescence. J Abnorm Child Psychol. 2016 Nov;44(8):1573–86. 10.1007/s10802-016-0132-1. 26846993

[31] Patalay P, Fonagy P, Deighton J, Belsky J, Vostanis P, Wolpert M. A general psychopathology factor in early adolescence. Br J Psychiatry. 2015 Jul;207(1):15–22. 10.1192/bjp.bp.114.149591. 25906794

[32] Caspi A, Houts RM, Belsky DW, Goldman-Mellor SJ, Harrington H, Israel S, et al. The *p* factor: one general psychopathology factor in the structure of psychiatric disorders? Clin Psychol Sci. 2014 Mar;2(2):119–37. 10.1177/2167702613497473. 25360393 PMC4209412

[33] Sallis H, Szekely E, Neumann A, Jolicoeur-Martineau A, Van IJzendoorn M, Hillegers M, et al. General psychopathology, internalising and externalising in children and functional outcomes in late adolescence. J Child Psychol Psychiatry. 2019 Nov;60(11):1183–90. 10.1111/jcpp.13067. 31049953 PMC6849715

[34] Laceulle OM, Vollebergh WAM, Ormel J. The structure of psychopathology in adolescence: replication of a general psychopathology factor in the TRAILS study. Clin Psychol Sci. 2015;3(6):850–60.

[35] Moore TM, Kaczkurkin AN, Durham EL, Jeong HJ, McDowell MG, Dupont RM, et al. Criterion validity and relationships between alternative hierarchical dimensional models of general and specific psychopathology. J Abnorm Psychol. 2020 Oct;129(7):677–88. 10.1037/abn0000601. 32672986 PMC7541771

[36] Cervin M, Norris LA, Ginsburg G, Gosch EA, Compton SN, Piacentini J, et al. The *p* factor consistently predicts long-term psychiatric and functional outcomes in anxiety-disordered youth. J Am Acad Child Adolesc Psychiatry. 2021 Jul 1;60(7):902–12. 32950650 10.1016/j.jaac.2020.08.440 PMC8109237

[37] Casey BJ, Cannonier T, Conley MI, Cohen AO, Barch DM, Heitzeg MM, et al. The adolescent brain cognitive development (ABCD) study: imaging acquisition across 21 sites. Dev Cogn Neurosci. 2018 Aug;32:43–54. 10.1016/j.dcn.2018.03.001. 29567376 PMC5999559

[38] Barch DM, Albaugh MD, Avenevoli S, Chang L, Clark DB, Glantz MD, et al. Demographic, physical and mental health assessments in the adolescent brain and cognitive development study: rationale and description. Dev Cogn Neurosci. 2018 Aug;32:55–66. 10.1016/j.dcn.2017.10.010. 29113758 PMC5934320

[39] Achenbach TM. Achenbach system of empirically based assessment (ASEBA). In: Cautin RL, Lilienfeld SO, editors. The encyclopedia of clinical psychology. Hoboken, NJ: Wiley; 2015, pp. 1–8. 10.1002/9781118625392.wbecp150.

[40] Kuznetsova A, Brockhoff PB, Christensen RHB. lmerTest package: tests in linear mixed effects models. J Stat Softw. 2017;82(13):1–26. 10.18637/jss.v082.i13.

[41] Brooks ME, Kristensen K, Benthem KJ, van MA, Berg CW, Nielsen A, et al. glmmTMB balances speed and flexibility among packages for zero-inflated generalized linear mixed modeling. R J. 2017;9(2):378. 10.32614/RJ-2017-066.

[42] Wen X, Shu Y, Qu D, Wang Y, Cui Z, Zhang X, et al. Associations of bullying perpetration and peer victimization subtypes with preadolescent’s suicidality, non-suicidal self-injury, neurocognition, and brain development. BMC Med. 2023 Apr 12;21(1):141. 10.1186/s12916-023-02808-8. 37046279 PMC10091581

[43] Paykel ES. Partial remission, residual symptoms, and relapse in depression. Dialogues Clin Neurosci. 2008 Dec 31;10(4):431–7. 10.31887/DCNS.2008.10.4/espaykel. 19170400 PMC3181895

[44] Fiorini G, Saunders R, Fonagy P, The Impact Consortium, Midgley N. Trajectories of change in general psychopathology levels among depressed adolescents in short-term psychotherapies. Psychother Res. 2023 Jan 2;33(1):96–107. 10.1080/10503307.2022.2040751. 35179082

[45] Lilienfeld SO. Comorbidity between and within childhood externalizing and internalizing disorders: reflections and directions. J Abnorm Child Psychol. 2003;31:285–91. 10.1023/A:1023229529866. 12774861

[46] Dalgleish T, Black M, Johnston D, Bevan A. Transdiagnostic approaches to mental health problems: current status and future directions. J Consult Clin Psychol. 2020;88(3):179. 32068421 10.1037/ccp0000482 PMC7027356

[47] Angold A, Weissman MM, John K, Merikancas KR, Prusoff BA, Wickramaratne P, et al. Parent and child reports of depressive symptoms in children at low and high risk of depression. J Child Psychol Psychiatry. 1987 Nov;28(6):901–15. 3436996 10.1111/j.1469-7610.1987.tb00678.x

[48] Cohen JR, So FK, Young JF, Hankin BL, Lee BA. Youth depression screening with parent and self-reports: assessing current and prospective depression risk. Child Psychiatry Hum Dev. 2019 Aug 1;50:647–60. 30737605 10.1007/s10578-019-00869-6 PMC6613974

