# Supplementary material for: The p factor outweighs the specific internalizing factor in predicting recurrences of adolescent depression
Source: Eur Psychiatry. 2024 Mar 1;67(1):e28. doi: 10.1192/j.eurpsy.2024.18 (PMC10988157; doi:10.1192/j.eurpsy.2024.18)
Supplement: Shu et al. supplementary material [file S092493382400018Xsup001.pdf]

SUPPLEMENTARY MATERIALS

Supplementary Table 1. *ABCD data release 3.0 variables used in current analysis* 1
Supplementary Table 2. *Generalized Linear Mixed Modelling results using full healthy control sample (n = 1597) after controlling for age, sex, race, and site* 2
Supplementary Figure 1. *Results of the p factor and the internalizing factor distinguishing between remitted depression patients and full sample healthy controls (n = 1597)* 3

Supplementary Table 1. *ABCD data release 3.0 variables used in current analysis*

Variable labels in current report	Variable labels in dataset	Scales in dataset
Sex	sex	pdem02
Age	interview_age	pdem02
Site	site_id_l	abcd_lt01
Race/Ethnicity	race_ethnicity	acspsw03

Supplementary Table 2. *Generalized Linear Mixed Modelling results using full healthy control sample (n = 1597) after controlling for age, sex, race, and site*

Metrics	Distinguish between remitted depression patients and HC															
	HC vs. G1								HC vs. G2							
	Baseline				2-year				Baseline				2-year			
	β	*SE*	*p*	p_{FDR}	β	*SE*	*p*	p_{FDR}	β	*SE*	*p*	p_{FDR}	β	*SE*	*p*	p_{FDR}
P factor	1.71	0.11	$<2\times10^{-16}$	7.66×10^{-52}	1.37	0.096	$<2\times10^{-16}$	3.63×10^{-46}	2.00	0.18	$<2\times10^{-16}$	1.48×10^{-29}	1.75	0.15	$<2\times10^{-16}$	7.45×10^{-31}
Internalizing factor	0.57	0.074	8.35×10^{-15}	2.51×10^{-14}	0.55	0.074	1.20×10^{-13}	2.41×10^{-13}	0.71	0.10	8.97×10^{-12}	1.35×10^{-11}	0.98	0.11	$<2\times10^{-16}$	9.83×10^{-19}

Note: *SE = Standard Error, p values in this table are the original p values before FDR correction.*

Supplementary Figure 1. *Results of the p factor and the internalizing factor distinguishing between remitted depression patients and full sample healthy controls (n = 1597)*

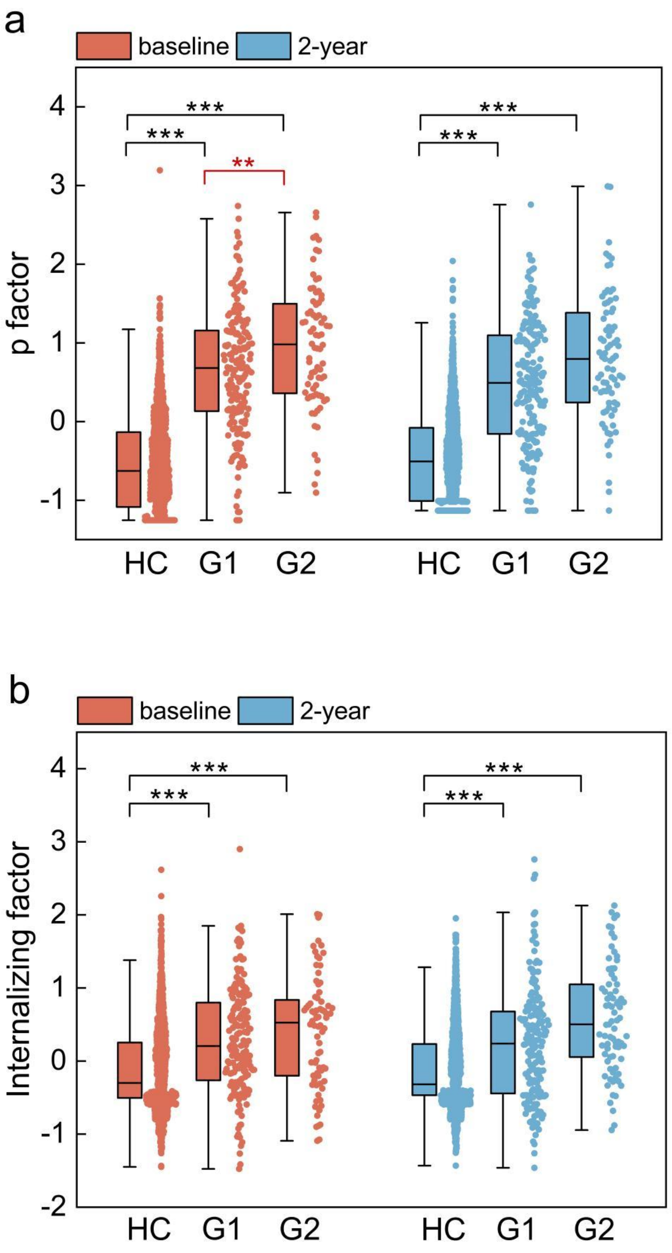

Note: *(a) represents the distribution of the p factor in each group at both baseline and 2-year-follow-up measurements. (b) represents the distribution of the specific internalizing factor in each group at both baseline and 2-year-follow-up measurements. *** = p <.001, ** = p < .01. HC = full Healthy Control group. G1 = Remission group, G2 = Recurrence group.*
